# How inter-state amity and animosity complement migration networks to drive refugee flows: A multi-layer network analysis, 1991–2016

**DOI:** 10.1371/journal.pone.0245712

**Published:** 2021-01-27

**Authors:** Justin Schon, Jeffrey C. Johnson

**Affiliations:** 1 University of Virginia, Charlottesville, Virginia, United States of America; 2 University of Florida, Gainesville, Florida, United States of America; Universitat de Valencia, SPAIN

## Abstract

What drives the formation and evolution of the global refugee flow network over time? Refugee flows in particular are widely explained as the result of pursuits for physical security, with recent research adding geopolitical considerations for why states accept refugees. We refine these arguments and classify them into explanations of people following existing migration networks and networks of inter-state amity and animosity. We also observe that structural network interdependencies may bias models of migration flows generally and refugee flows specifically. To account for these dependencies, we use a dyadic hypothesis testing method—Multiple Regression- Quadratic Assignment Procedure (MR-QAP). We estimate MR-QAP models for each year during the 1991–2016 time period. K-means clustering analysis with visualization supported by multi-dimensional scaling allows us to identify categories of variables and years. We find support for the categorization of drivers of refugee flows into migration networks and inter-state amity and animosity. This includes key nuance that, while contiguity has maintained a positive influence on refugee flows, the magnitude of that influence has declined over time. Strategic rivalry also has a positive influence on refugee flows via dyad-level correlations and its effect on the structure of the global refugee flow network. In addition, we find clear support for the global refugee flow network shifting after the Arab Spring in 2011, and drivers of refugee flows shifting after 2012. Our findings contribute to the study of refugee flows, international migration, alliance and rivalry relationships, and the application of social network analysis to international relations.

## Introduction

Refugee flows have become a source of global concern. Origin countries fear lost human capital, lost legitimacy for their governments, and the possibility that political opponents will establish bases outside their reach [1–3]. Destination countries fear the tensions that come with shifts in their demographic balances and the diffusion of conflict and terrorism [4, 5]. Governments in many destination countries have therefore implemented measures to secure their borders and prevent immigration [6, 7]. Yet, refugee flows may follow specific patterns due to networks that exist within the international system [8, 9]. These networks may be too powerful for individual countries to effectively block refugee flows. This leads to the question: What drives the formation and evolution of the global refugee flow network over time?

To answer this question, we contend that it is valuable to examine the many overlapping networks that form the international system [10–12]. Disparities in well-being are important, but they do not tell the whole story [13]. Networks of international trade, international migration, alliances, rivalry, physical distance, and other factors combine to form the structure of motivations and opportunities that drive global refugee flows [8, 14–19].

Our analysis focuses on the post-Cold War 1991–2016 time period. As migration research such as Orchard [20] observes, the post-Cold War time period has experienced multiple phases in refugee flow patterns. Large refugee flows during the early 1990s that resulted from episodes such as the breakup of Yugoslavia, Siad Barre’s removal from power in Somalia, the Taliban’s takeover of Afghanistan, and mass killing and genocide in Rwanda and Burundi were met with the exhaustion of American and other Western governments from hosting refugees during the Cold War [20]. Neighboring countries such as Kenya, Iran, Pakistan, Tanzania, and the Democratic Republic of the Congo welcomed refugees, but their hospitality progressively waned by the end of the 1990s [21–23]. In the early 2000s, governments around the world rapidly increased the amount of border walls and obstacles to refugee flows and international migration [7, 24–26]. By the end of the 2000s, new refugee flows had fallen to relatively low levels. In 2011, the Arab Spring sparked renewed surges of refugees. Governments fell and civil wars began across Tunisia, Libya, Egypt, Syria, Yemen, and Iraq [27]. These developments destabilized other countries as well. Libya’s collapse contributed to flows of weapons and fighters into Mali, Niger, Chad, Sudan, and other countries in the African Sahel [28]. While these developments primarily led to internal displacement in 2011, refugee flows increased substantially starting in 2012. The 2012–2016 time period marks the clearest coherent phase of refugee flows after the Cold War, so we believe that this is a particularly valuable time period to analyze.

We hypothesize that inter-state geopolitical networks, specifically networks of amity and networks of animosity, and international migration networks are the most important types of factors for explaining refugee flows. While the growing field of refugee studies often attempts to explain refugee flows, like international migration, through a focus on international migration networks [29], we contend that geopolitical factors are also important. Moorthy and Brathwaite [30] and Jackson and Atkinson [31] show that power competition in the form of rivalry has a significant positive effect on refugee flows. These networks of animosity may treat refugee flows as valuable foreign policy tools [2, 32]. On the other hand, networks of amity may show refugees where they will find welcoming hosts.

## Data and methods

Quantitative network analysis can help disentangle which of these networks are important drivers of refugee flows. We estimate one Multiple Regression- Quadratic Assignment Procedure (MR-QAP) model for each year from 1991–2016 in order to identify the variables that are significantly related with refugee flows. MR-QAP is a dyadic hypothesis testing method that accounts for dyad-level correlations and interdependencies across dyads, addressing known weaknesses of traditional regression analysis [14, 33].

MR-QAP models require input variables to be in the form of square matrices. For the 1991–2016 time period, we therefore use 195 x 195 square matrices for each variable. Prior to 2011, these square matrices are smaller. For example, South Sudan became a country in 2011, so we work with 194 x 194 square matrices for 2010. With these matrices, the first step is to estimate an ordinary least squares (OLS) regression. Due to concerns about the sensitivity of MR-QAP to collinearity, we checked Variance Inflation Factors (VIFs) for all of our models [34]. In all cases, our models did not show evidence of collinearity.

Then, we use the estimated t-statistic and compare it to a distribution of t-statistics. That distribution is calculated through 1000 Monte Carlo simulations where rows and columns of the dependent variable are shuffled, while maintaining row-column combinations. Our quadratic assignment procedure uses the double semi-partialing permutation method, which is the most robust permutation approach [34]. This process isolates dyad-level correlations. It is then possible to estimate a p-value comparing the observed t-statistic with the simulated distribution of t-statistics [35, 36]. We use the netlm function from the statnet package in R for this analysis [37]. Since the software computes the MR-QAP-adjusted p-value but not an adjusted t-statistic, we use the adjusted p-values and degrees of freedom for each year to calculate our own MR-QAP-adjusted t-statistics.

We save the MR-QAP-adjusted t-statistics and OLS t-statistics and then analyze the annual variation in those t-statistics. For the MR-QAP-adjusted t-statistics, we use k-means clustering analysis to classify our variables into 3 groups and our years into 3 groups. The clustering by variables compares the annual variation in MR-QAP-adjusted t-statistics across years. By contrast, the clustering by years compares the annual variation in MR-QAP-adjusted t-statistics across variables. We estimate our clusters from distance matrices based on Pearson’s correlation coefficients. Then, we use multi-dimensional scaling (MDS) analysis to visualize these classifications.

Table 1 displays the variables and data sources used for the analysis. Replication materials will be made available online. Our dependent variable, *Refugee Flows*, comes from refugee data from the United Nations High Commissioner for Refugees (UNHCR). This resource includes dyadic refugee stocks from 1975–2016, excluding Palestinian refugees since they fall under the mandate of the United Nations Relief and Works Agency (UNRWA). Before 1990, a large portion of UNHCR’s refugee data is not actually dyadic, so we would advise researchers to only use UNHCR’s monadic data if they wish to conduct analysis on refugee population data pre-1990. We calculate the first difference of refugee stocks to obtain net refugee flows. We then replaced missing values with zero. We also followed existing standard practice and replaced negative net refugee flow values with zero. Our measure of refugee flows should be considered as an undercount of flows, since it does not include short-term movements or return movements. This is, however, the best available measurement of refugee flows.

**Table 1:** Variable descriptions.

Variable	Description	Source
*Dependent Variable*		
Refugee Flows	First difference in dyadic refugee stocks **(Flows from country i to country j)**	UNHCR
*Independent Variables*		
Security Gradient	Difference in political terror between destination and origin country **(Gradient)**	Political Terror Scale
Wage Gradient	Destination country GDP per capita minus origin country GDP per capita **(Gradient)**	World Development Indicators
Immigrant Population	Total migrant stock in 1990, 2000, or 2010 **(Flows from country i to country j)**	World Bank Bilateral Migration Matrix
Prior refugee flows	One year lag of refugee flows **(Flows from country i to country j)**	UNHCR
International trade	Magnitude of international trade **(Flows from country i to country j)**	Correlates of War
Regime Type Gradient	Difference between destination and origin country regime type **(Gradient)**	V-DEM
Alliance	Existence of an alliance of mutual defense **(Undirected dyad)**	ATOP version 4.01
Arms Flows	Quantity of arms flows in SIPRI units **(Flows from country j to country i)**	SIPRI Arms Transfers Database
Strategic Rivalry	Existence of strategic rivalry **(Undirected dyad)**	Thompson & Dreyer (2011); updated by authors through 2016
Contiguity	Dichotomous indicator of land or water contiguity **(Undirected dyad)**	Correlates of War

Next, we add our independent variables. While we only discuss the results for full models with all independent variables, bivariate regressions yield consistent results. Our variable *Prior Refugee Flows* is a measurement of net refugee flows in the previous year. Our measurement of immigration, *Immigrant Population*, comes from the World Bank’s data on bilateral international migration. It measures dyadic immigrant stocks as of 1990, 2000, or 2010. We use the most recent prior immigrant population measure. We then used Benjamin Graham’s and Jacob Tucker’s data repository to obtain data on GDP per capita from the World Bank’s World Development Indicators database (*Income Gradient*), the Varieties of Democracy (V-DEM) additive polyarchy measure of regime type (*Regime Type Gradient*), and the Political Terror Scale (PTS) index value for each country (*Security Gradient*) [38]. From these monadic variables, we created gradient matrices to capture the difference in these values between destination and origin countries. For *Security Gradient*, we used values coded from Amnesty International reports. When those were missing, we used values coded from State Department reports. This yielded a variable with zero missing values for 2015. For 2011–2014, we used values coded from Human Rights Watch reports when there were missings for Amnesty International and the State Department. For remaining missing values, we imputed a value of zero. For *Regime Type Gradient*, we replaced missing dyad gradient values with zero. For *Income Gradient*, we also replaced missing dyad gradient values with zero.

Our *Security Gradient* and *Income Gradient* variables only go through 2015, so we used those values for our 2016 models as well. Our measurement of dyadic arms flows, *Arms Flows*, comes from annual dyadic data from the Stockholm International Peace Research Institute. We replaced missing values of *Arms Flows* with zero. Our measure of international trade, *Trade*, comes from the Correlates of War project [39]. This variable only goes through 2014, so we used the 2014 values for models of refugee flows in 2015 and 2016.

Our measure of contiguity (*Contiguity*) also comes from the Correlates of War project [40]. Contiguity is a critical geographic variable because it captures the increased familiarity that people have with neighboring countries, higher likelihood of shared languages and customs, and easier and cheaper transportation options [29, 41, 42].

Our measure of alliances of mutual defense (*Alliance of Defense*) comes from the Alliance Treaty Obligations and Provisions Project (ATOP) [43]. Our rivalry measure, *Strategic Rivalry*, comes from the rivalry dataset created by William R. Thompson [16, 44]. The strategic rivalry dataset codes rivalries through 2010, so we updated the coding of strategic rivalries through 2016. Details about the updated coding of strategic rivalries will be posted on the lead author’s personal website.

## Results

**Fig 1:**
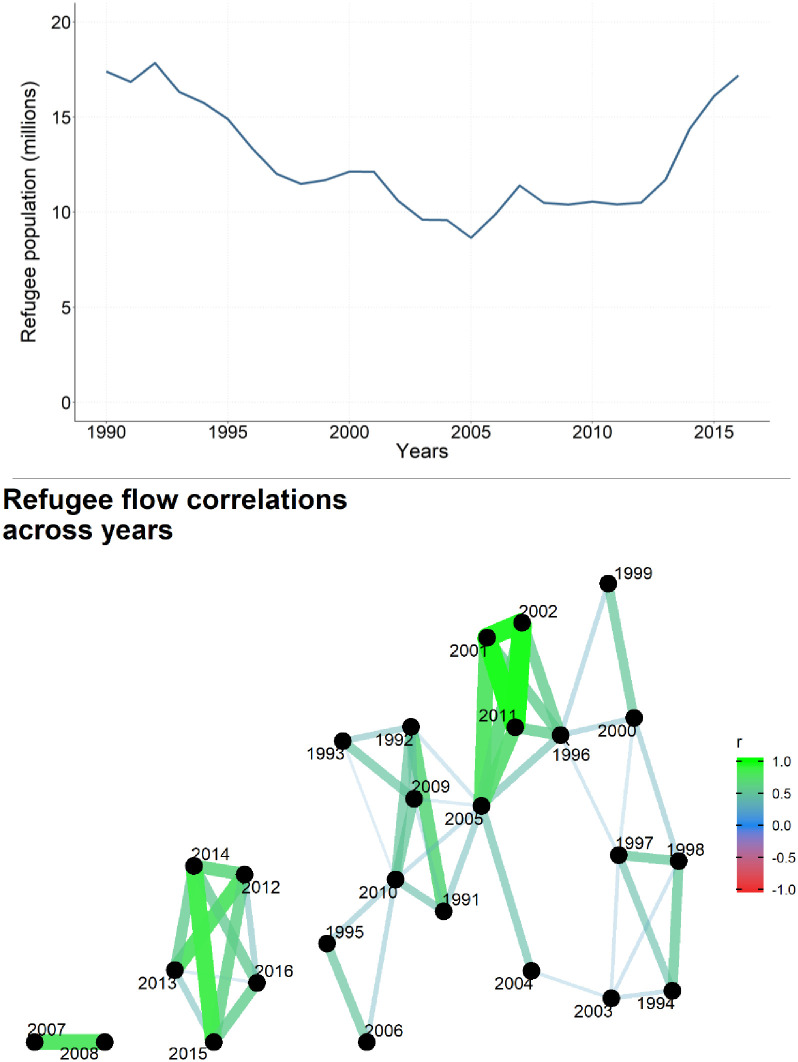
World refugee population, excluding Palestinian refugees (top) & refugee flows, correlated across years (bottom).

Fig 1 describes post-Cold War refugee flows. At the end of the Cold War, the breakup of the Soviet Union and Yugoslavia, collapse of the Somali state, and other conflicts drove the world’s refugee population over 15 million. As the 1990s proceeded though, conflicts progressively ended. The loss of sponsorship for opposing sides from the United States and Soviet Union removed fuel that had been allowing many wars to persist [45]. This period of conflict termination coincided with a decline in the world’s refugee population. Fig 1 shows that the global refugee population progressively fell from 1990 to 2005. In addition to the global refugee population falling as conflicts terminate, the refugee population also increased when new conflicts began. From 2005–2007, the world’s refugee population increased, before remaining relatively constant until 2012. During the 2012–2016 time period, the world refugee population under UNHCR’s mandate increased substantially, from roughly 10.5 million to 17 million refugees.

This explosion of refugee flows corresponds with the violent aftermath of the Arab Spring, which included the fall of governments in Tunisia, Egypt, Yemen, and Libya, as well as civil wars in Syria, Libya, and Yemen [27]. These developments destabilized other countries not directly involved in the Arab Spring as well. Libya’s collapse flooded the African Sahel with fighters and weapons [46]. Syria’s civil war contributed to civil war in Iraq [47]. These challenges compounded existing issues with the founding of South Sudan in 2011, Somalia’s 2011 drought that triggered more refugee flows than Somalia had produced since the early 1990s, and the straining of host communities in Iran and Pakistan to host millions of Afghan refugees. Political instability did not really start affecting refugee flows until 2012 because its main effect initially was to fuel internal displacement in 2011. Beginning in 2012, however, the new phase of conflict and migration activity increased refugee flows.

The increased activity in the global refugee flow network from 2012–2016 created substantial international concern. In addition to a rapidly growing worldwide refugee population, the world also experienced refugee flows through different sets of directed dyads. In Fig 1, we illustrate this with a correlation network. Our correlation network defines weighted ties between each year’s set of directed dyads based on the Pearson’s correlation coefficient. For clarity in our visualization, we exclude ties between years that have a correlation coefficient less than 0.2. As Fig 1 shows, all correlation coefficients were greater than 0.2. Refugee flows from 2012–2016 were highly correlated at the directed dyad level (with 2014–2015 having the highest correlation coefficient of 0.86), whereas all other years except for 2007 and 2008 formed a large and indistinct jumble. These trends in the aggregate and at the directed dyad level suggests that something new was happening. The 2012–2016 time period was a new phase in global refugee flow patterns.

**Fig 2:**
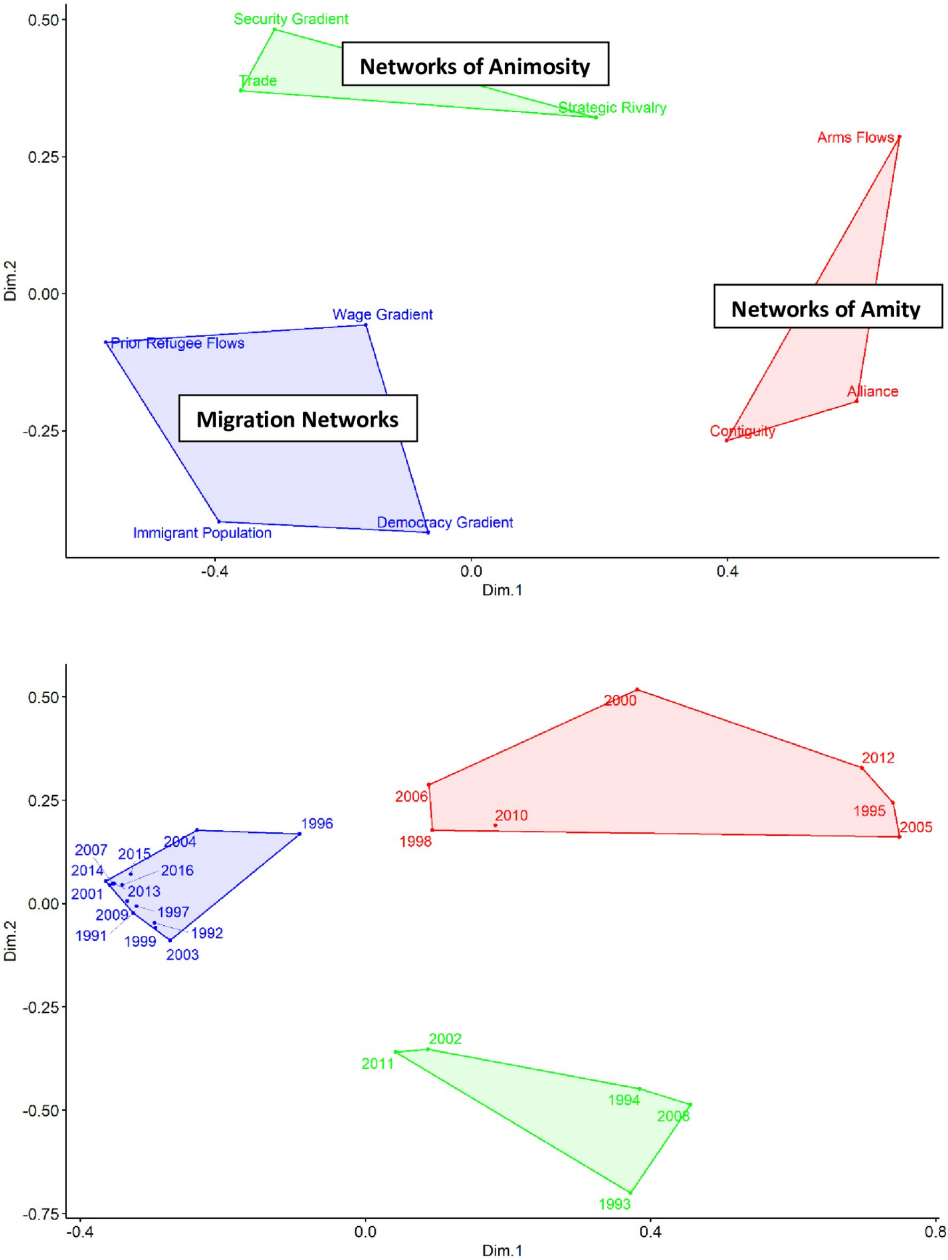
MDS variables (top) & years (bottom).

Fig 2 displays MDS plots of the k-means clustering analysis that build on this descriptive insight. Cluster analysis of variables shows that they fit together into three categories: 1) *Strategic Rivalry*, *Security Gradient*, and *Trade*; 2) *Alliance*, *Arms Flows*, and *Contiguity*; and 3) *Prior Refugee Flows*, *Immigrant Population*, *Wage Gradient*, and *Democracy Gradient*. These groupings align with the three categories that we discussed in the Introduction: migration networks, networks of animosity, and networks of amity. Here, there is some support for the view that *Wage Gradient* and *Democracy Gradient* matter for the creation of migration networks, and then *Prior Refugee Flows* and *Immigrant Population* matter for the maintenance of migration networks [48–50]. Then, *Arms Flows* and *Alliance* fit well within the category of networks of amity. The inclusion of *Contiguity* in this category suggests that contiguity increases refugee flows via a “familiarity breeds friendship” effect, rather than by contributing to the creation or maintenance of migration networks through a “familiarity breeds contempt” effect.

**Fig 3:**
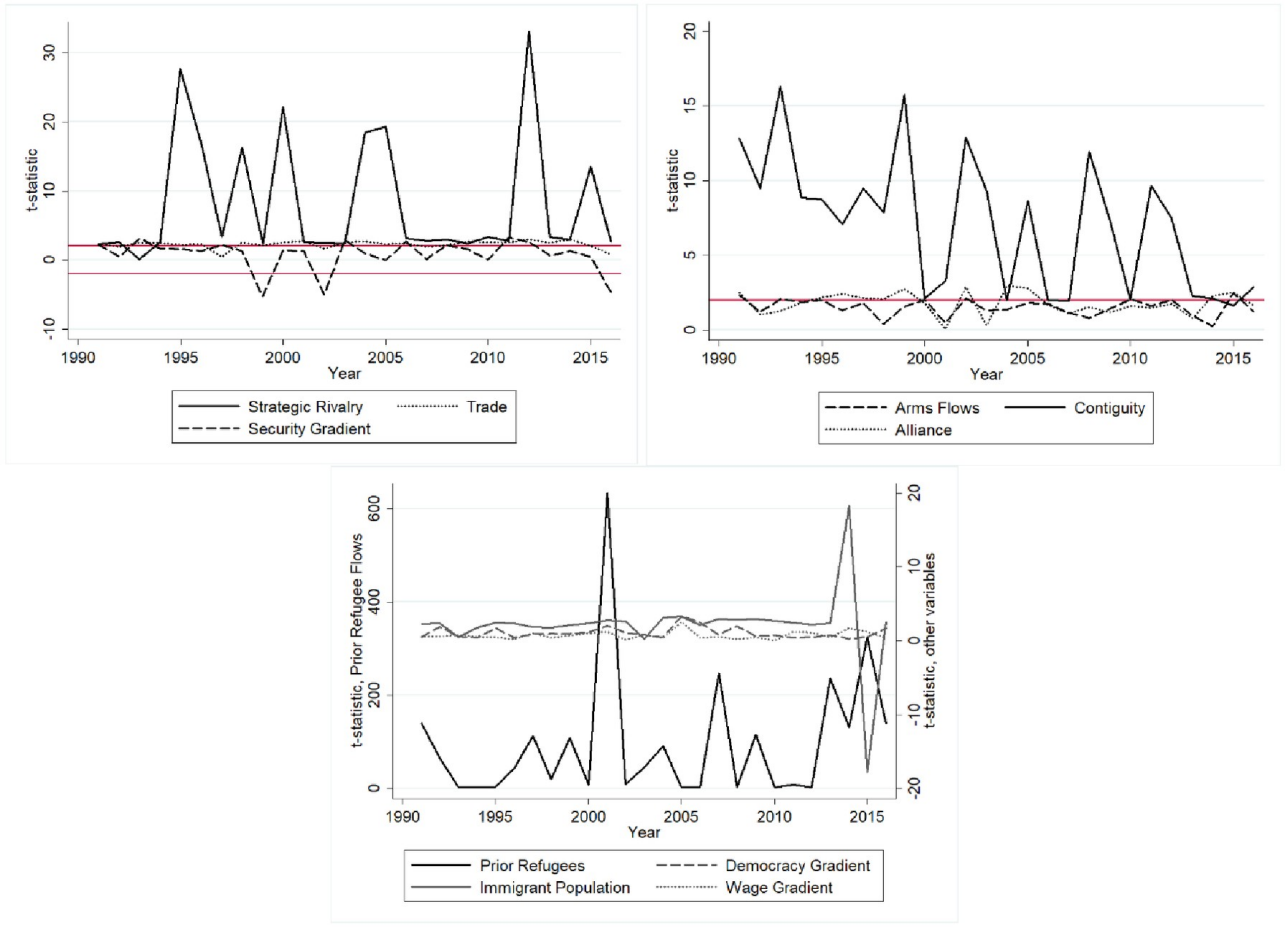
MR-QAP-adjusted t-statistics networks of animosity (top left); networks of amity (top right); migration networks (bottom); t-statistic reference lines at +/- 2.

**Fig 4:**
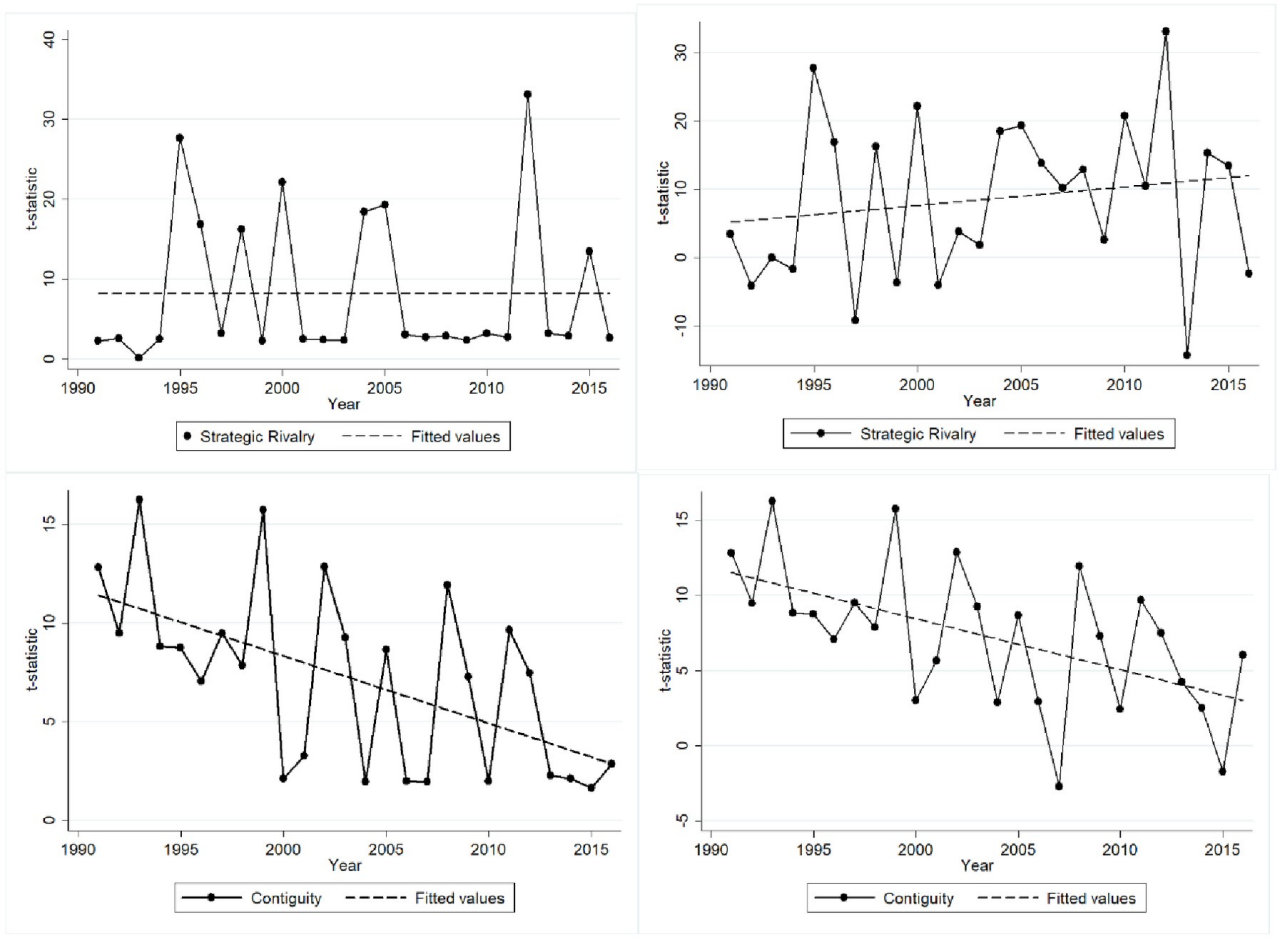
Rivalry & contiguity with fitted lines (MR-QAP results top-left and bottom-left, OLS results top-right and bottom-right).

The networks of animosity category functions differently from the networks of amity category, supporting the view that friendship and hostility are alternative pathways in international relations [51]. Fig 3 shows that rivalry becomes more important in driving refugee flows when trade becomes less important and relative safety becomes more important. Readers should remember when interpreting Fig 4 that due to how *Security Gradient* is coded, negative t-statistics actually mean that there are higher refugee flows when the destination country is safer than the origin country.

Meanwhile, there are some years where refugee flows actually tend to move towards countries that are *less* safe than the origin country (e.g.- 1993, 2003, 2011). *Trade* generally has a positive and significant relationship with refugee flows, but its clustering into the “networks of animosity” category is unexpected. For most of the 1991–2016 time period, *Trade* has a fairly constant relationship with refugee flows at around +2. Then, from 2013–2016 it fluctuates in the opposite direction from *Strategic Rivalry*.

Fig 3 also displays the t-statistics for migration networks. It is clear that *Prior Refugee Flows* has t-statistics that are substantially larger than *Immigrant Population*, *Wage Gradient*, and *Democracy Gradient*. *Immigrant Population* also tends to have significant and positive relationships with *Refugee Flows*. *Wage Gradient* is generally not significant. *Democracy Gradient* has a positive and significant relationship with refugee flows in some years, but it also does not generally appear to be important. Moreover, the inclusion of *Democracy Gradient* in the “migration networks” category suggests that this variable matters for its role in facilitating the creation of international migration networks. Like the “networks of animosity” category, the “migration networks” category has an important shift during the 2013–2016 time period. Here, the t-statistics for *Prior Refugee Flows* and *Immigrant Population* fluctuate in clear opposite directions from 2013–2016.

The third set of t-statistics in Fig 3 is for networks of amity. *Alliance* and *Arms Flows* are usually not statistically significant, but there are several years where *Alliance* has a positive and significant relationship with *Refugee Flows*. *Contiguity*, meanwhile, is positive and significant for every year except 2015. Again, 2013–2016 exhibits some different dynamics. Here, *Alliance* and *Contiguity* suddenly fluctuate in opposite directions, with *Contiguity*’s t-statistic generally shrinking as *Alliance*’s t-statistic grows. This suggests that there may be a saturation effect in contiguous states that led to onward migration, which was influenced by geopolitical considerations.

There are important temporal dynamics for *Contiguity* and *Strategic Rivalry* that merit additional analysis. For this purpose, we refer to Fig 4. All plots include a fitted line in order to clarify the temporal trend. For both variables, we include one plot with the time series of MR-QAP-adjusted t-statistics and one plot with the time series of OLS t-statistics. From these plots, it appears that *Strategic Rivalry* has a growing effect over time in the OLS results, whereas its effect is relatively constant over time in the MR-QAP results. *Contiguity* does not have notable differences between the t-statistics from MR-QAP and OLS. This distinction suggests that *Strategic Rivalry* may influence the structure of the global refugee flow network, not just the magnitude of individual refugee flows. Our results do not allow us to specify the specific network structures in the global refugee flow network that are influenced by strategic rivalry, but the observation that network structures may be influenced by dyadic relationships is important in and of itself. Meanwhile, *Contiguity* has a declining effect on *Refugee Flows* over time. In other words, since the end of the Cold War, refugees have been moving further away from their origin countries.

We also examine our observation that the drivers of refugee flows appear to shift during the 2013–2016 time period. In Fig 1, we observed that the directed dyads of the global refugee flow network are strongly correlated from 2012–2016. This is also a period where the global refugee population rose substantially. Therefore, the world was experiencing a surge in refugee flows and that surge was occurring through a new set of directed dyads. Fig 2 shows that when we cluster t-statistics over years, that surge was also being driven by a new set of factors. When t-statistics for the 1991–2016 models are clustered into three groups over years, there is a lot of noise. One important distinction is that the years 2010–2012 cluster into different groups than the years 2013–2016. Our year clusters do not just reflect when refugee populations were rising, falling, or flat, since there is not a coherent pattern for how years cluster before 2013 in Fig 2. These observations all indicate that something important changed starting in 2012. We cannot specify one trigger with certainty, but as we discussed earlier, the Arab Spring and related instability in 2011 is a plausible culprit. If the Arab Spring were actually unrelated to this shift in refugee flow patterns, our finding remains that something changed during the early 2010s.

## Discussion

In this paper, we have demonstrated the value of explaining refugee flows as a multi-layer network. An analysis that incorporates multiple overlapping networks allows us to account for many kinds of network dependencies. Our analysis describes the evolution of the global refugee flow network since the end of the Cold War. Considering correlations between the sets of existing refugee flow directed dyads in each year, we observe a noisy process from year to year. The 2012–2016 time period, however, stands out as a period with highly inter-correlated refugee flows across years. We applied MR-QAP and learned that refugee flows vary based on variation in factors related to international migration networks, networks of animosity, and networks of amity.

There are several possibilities for future research. It would be valuable to use these macro-level findings to guide further research on refugee flow ego networks (e.g., focus on a single country’s flows to unpack spatial and temporal dynamics) and on emerging drivers of refugee flows like climate change. Like the climate-conflict research program [52], climate-induced migration research argues that the role of climate and environmental factors is likely to increase substantially in the coming decades. In addition, we find evidence for a role of inter-state amity, as well as animosity, in facilitating refugee flows. New research should follow-up on our findings and consider the role of alliances and rivalries. Since rivalry may influence network structures of the global refugee network, not just dyadic refugee flows, future research could also explore which network structures in the refugee flow network change due to rivalry. Finally, the shift in refugee flow patterns after 2011 suggests that additional research on the Arab Spring and other key events from the early 2010s could contribute to broad understandings of changes in the international system, beyond changes in the Middle East alone.

## Supporting information

S1 File (ZIP)
